# Multi-Indicator Intelligent Monitoring of Clinical Observations to Reduce Cesarean Section

**DOI:** 10.1155/2021/8139200

**Published:** 2021-11-24

**Authors:** Shasha Xie, Wei Dong, Yeting Liu, Haixiao Gao, Dan Zhang

**Affiliations:** ^1^Affliated Xingtai People's Hospital of Heibei Medical University, Xingtai 054000, Hebei, China; ^2^The Third Hospital of Xingtai City, Xingtai 054000, Hebei, China; ^3^The First Affliated Hospital, Xingtai Medical College, Xingtai 054000, Hebei, China

## Abstract

In order to analyze multi-index monitoring and the effect of reducing cesarean section, this paper selects March 2018 and March 2019 in two obstetrics and gynecology hospitals, referred to as hospital A and hospital B. As research objects, 313 pregnant women were divided into multi-index group and conventional group, while analyzing various indicators of each group of cesarean collection. The results show that the total CNAXE rate was 48.10% and 39.29%, respectively, for 2018 and 2019, respectively, and the cesarean section of the conventional group was 65.75% and 63.64%. By contrasting data of multi-index group and conventional group, hospital B differences were significant (*P* < 0.05), and hospital A difference was extremely significant (*P* < 0.01). In Cesarean section, obstetric sectors can help maternal treatment strategies by monitoring a series of related indicators for maternal to reduce Cesarean section and improve prognosis.

## 1. Introduction

In obstetric clinical treatment, cesarean section (CS) is a more common surgical method in the clinical treatment of obstetrics, is an effective means of solving difficulties, and has an important role in the treatment of high-risk pregnancy. However, as the ratio of Cesarean section continues to rise, the long-term short-term adverse prognosis of Cesarean section is increasingly prominent; Cesarean section is a scientific medical measure, and its role is to promote people's health; in order to ensure this, the goal requires reasonable use of Cesarean section in the actual population. If cesarean section is misused and abused, it will not only affect the efficiency of medical resources, but also increase the economic burden of pregnant women, but also have a great impact and risk on the health of mothers and infants. According to World Health Organization, the Sampling of Asian Country 2007–2008 was published in the “Willow Knife.” The total percentage of Cesarean section in Asia is 27.3%, with China contributing up to 46.2%. The percentage of Cesarean section in USA was only 5.5% in the 1970s, which is the same as that of China in the same period, and it has risen to 25% in the late 1980s. It was nearly 5 times in the 1970s; the percentage of Cesarean section in UK was 6%–8%, and, with slow rise, in the 1990s, the Cesarean section exceeded 20% of the Cesarean market rate; so far, Brazil, South America, has been the highest in the world, reaching 50.8% in 1994, and in 1996 the ratio was still as high as 36.4%. According to relevant studies, if the probability of the pregnant woman's Cesarean section is greater than 30%, the harm of the mother and child is increasing when using the Cesarean section. According to relevant reports, the United States had 2.2 people in the mortality rate of pregnant women in Cesarean section from 2000 to 2006, and the mortality rate of vaginal delivery is 0.2 people in every 100,000 people. Therefore, compared with the vaginal childbirth, it is not very safe. There is a possibility of causing a variety of complications to pregnant women using Cesarean section. For example, in surgery, anesthesia is unexpected, due to the bleeding, infection, and supine of low blood pressure in the uterine contraction, as well as the syndrome of low blood pressure; in surgery, the pregnant woman is prone to puerperium infection, postpartum hemorrhage (PPH), low back pain, lactation, pelvic or lower extremitic thrombosis, advanced postpartum bleeding, endometriosis, endometriosis, and so forth. Pregnant women are prone to placenta previa, placental detachment, ectopic pregnancy, and uterine injury. Compared with newborns from normal childbirth, infants who are born by Cesarean section are prone to wet lungs, hospitality premature birth, neonatal pulmonary transparent film disease, newborns jaundice, low immunity, childhood sensory issues and disorders, and bronchial asthma. Such a series of complications increase the proportion of newborns admitted to NICU. As a qualified medical staff, we should strictly follow the instructions of Cesarean section surgery, while the public should also understand the advantages and disadvantages of Cesarean section, try as possible the process of natural delivery, and reduce unnecessary Cesarean sections; in particular, the surgical indications for Cesarean section must be strictly grasped [1].

## 2. Literature Review

In recent years, due to the increase in Cesarean section, the number of maternal women with “scar uterus” has increased year by year. According to relevant reports, the repetitive Cesarean section rate of pregnant women in China has reached 92.5%. According to relevant foreign literature, pregnant women can perform vaginal test production after Cesarean section, and success rate can reach 60%–80%. With the changes in medical environment and the implementation of the family planning policy advocated by China, the proportion of Cesarean section of “nonmedical indications” is increasing, because the pregnant women and their families have insufficient understanding of Cesarean section. Forever scar uterus will lead to subsequent pregnancy pathological conditions and second Cesarean section (SSC) (duplicate Cesarean section, DSC), front placenta (Placenta Previa), PP), placental implantation, and so forth, as well as the emergence of serious complications that endanger the safety of the mother and children. Recently, how to safely reduce the Cesarean section has become an important topic of the current obstetrics and requires multidisciplinary actions, including research on science and anesthesiology. The famous medical researcher Zhang L. and colleagues have carried out a study on past Cesarean section. It is believed that the rise of Cesarean section is the culprit of women in various complications, such as consolidating hypertension or concurrent diabetes. Therefore, it is suggested that women choose natural delivery in the actual mode of delivery [2]. The famous medical researchers Gassama O. and colleagues believe that the change in Cesarean section happens with the changes in medical technology, socioeconomic development, and pregnant women's understanding of childbirth [3]. Therefore, the study of the indication of Cesarean section cannot be just in one year; there should be a valid analysis of multiyear data. In the case of foreign Cesarean section research, China started late in Cesarean section. Our department also started research and control of Cesarean section in 2006. Some scholars have carried out good studies on Cesarean section; it is considered that the actual investment in Cesarean section today is mainly concentrated in the repeated pregnancy after Cesarean Section, RCS, huge fetus (Macrosomia), and less amniotic fluid (Oligohydramnios and Fetal Distress, Social Factors, Social Factors, etc.), and related medical institutions should be analyzed and have certain effective measures for corresponding indications. This can really reduce the proportion of higher-tight-bedroom proportion. In summary, due to the contrarity of Cesarean section in the past few years, the maternal Cesarean section has increased rapidly, it is difficult to control, and the Cesarean section is constantly expanding. The main reason for this phenomenon is not just a medical problem or social problems. In this regard, we should analyze the main causes of this phenomenon and conduct a reasonable propaganda for the true indications of Cesarean section and bad disadvantages [4]. On the basis of current research, this paper selects March 2018 and March 2019 in two obstetrics and gynecology hospitals, referred to as hospital A and hospital B. 313 pregnant women were divided into multi-index group and conventional group, while analyzing various indicators of each group of cesarean collection. The results show that the total CNAXE rate was 48.10% and 39.29%, respectively, for 2018 and 2019, respectively, and the cesarean section of the conventional group was 65.75% and 63.64%. By contrasting data of multi-index group and conventional group, hospital B differences were significant (*P* < 0.05), and hospital A difference was extremely significant (*P* < 0.01). In Cesarean section, obstetric sectors can help maternal treatment strategies by monitoring a series of related indicators to reduce Cesarean section and improve prognosis.

## 3. Data and Methods

### 3.1. General Information

313 cases of pregnant women from March 2018 to December 2019 in hospital A of obstetrics and gynecology and hospital b of obstetrics and gynecology were selected, including 152 cases divided into 79 cases in multi-independent group and 73 cases in regular group. The age is 22–39 years (average 27.41 ± 3.53); the pregnancy is 36–42 weeks, with average of 38.39 ± 4.35 weeks. Hospital A had 161 cases: 84 cases of multi-indicator group and 77 cases of regular group, with ages of 20 to 38 years (average 27.14 ± 2.85) and pregnancy of 37–42 weeks (average 39.30 ± 1.38). The cases of the two hospitals had no history of internal surgery and pelvic stenosis, pregnancy complications, and so forth in the week before birth or before labor, and B-ultrasound examination and fetal heart rate monitoring were performed.

### 3.2. Checking Method

(1) The grayscale B-ultrasound (3.5–5.0 MHz, the vaginal probe frequency is 6.5 MHz) and the color Doppler ultrasonic diagnostic instrument (the probe frequency is 3.5 MHz), respectively, are used, and the selected pregnant woman is examined. In the multi-indicator group, pregnant women take the stone frame and put the vaginal probe plug, the head is applied to the pocket and then put into the vagina, and then the double top diameter (BPD), head circumference (HC), discontinuation (AD), abdominal circumference (AC), femur length (FL), femoral skin fat thickness (FTH), fetus, amniotic fluid, cervical length (CVL), and uterine scars thickness are measured. In the conventional group, pregnant women are subjected to an end-length position and BPD, FL, fetus, and amniotic fluid index are measured. (2) Both groups use the fetal heart monitor to conduct half-hour monitoring of the pregnant women to obtain NST scoring materials [5–7].

### 3.3. Measurement Indicators

#### 3.3.1. Predicting a Huge Indicator

The multi-index group is predicted by BPD ≥9.5 cm, FL ≥ 7.5 cm, and AC ≥ 37 cm, and the conventional group is only predicted with BPD ≥9.5 cm and FL ≥ 7.5 cm.

#### 3.3.2. Umbilical Inspection of the Neck

According to the standard of Obstetrics and gynecology: abdominal or vaginal B-ultrasound examination, there are obvious indentation on the skin around the umbilical cord. Or color Doppler examination showed umbilical cord blood flow signal in the longitudinal section of fetal neck, and umbilical cord blood flow around fetal neck in the transverse section.

#### 3.3.3. Fetal Distress Indicator

The diagnostic criteria of “obstetrics and gynecology” were used, and Apgar score was used. The infants were scored at 1, 5, and 10 minutes after birth. 0–3 points were severe asphyxia, and 4–7 points were mild asphyxia

#### 3.3.4. Cervical Maturity Indicator

According to the literature, it is defined as the cervidal length of 30 mm.

#### 3.3.5. Uterine Scar Thickness

The measurement method of “Ultrasonic Diagnosis” of Utility Obstetrics and Gynecology is used in the edge of the fetus to the fetus. The distance > 30 mm is standard [8–10].

### 3.4. Statistical Method

The count data is used in *χ*2 test.

## 4. Results

Comparison of the giant childhood delivery outcomes in each group of hospital is shown in Table 1. Comparison of the endings of the two hospitals in each group is shown in Table 2. Comparison of the fetches of the fetuses of the two hospitals is shown in Table 3.

### 4.1. Determination of Cervical Maturity

Hospital B in the multi-indicator group uses vaginal B-ultrasound to measure the length of 79 pregnant women, including 25 cases of cervical length ≥ 30 mm, 18 cases of Tongzhani (72.00%), 7 cases of waters <30 mm, all vaginal Childbirth (28.00%); hospital A in the multi-independent group measures the length of the cervix in 84 pregnant women, with 31 cases having a length ≥30 mm, 26 cases of cesarean section, accounting for 83.87%; cervical length  <  30 mm in 5 cases, all vaginal delivery, accounting for 16.13% [11–13].

### 4.2. Scar Thickness of Uterus

There were 12 cases of the 79 cases in the multi-index group of hospital B suffering from scar uterus: 5 cases had scar thickness ≤30 mm, all Cesarean section (41.67%), and 7 cases had scar thickness >30 mm, all of which were vaginal delivery (58.33%). Among the 79 cases in the multi-index group of hospital B, 12 cases had scar uterus, 5 cases had scar thickness ≤30 mm, all of which were cesarean section (41.67%), and 7 cases had scar thickness >30 mm, and all of which were vaginal delivery (58.33%).

### 4.3. Comparison of Delivery Outcomes in Different Fetal Positions

Comparison of the outcomes of the two hospitals in various groups of orientation is shown in Table 4.

### 4.4. Comparison of Childbirth Endings

The percentages of Cesarean section in the two hospitals were 48.10% and 39.29%, respectively, and the percentages of Cesarean section in the conventional group were 65.75% and 63.64%, respectively, and there is no significant difference between multi-index group and conventional group in the two hospitals. In a comparison of Cesarean section, hospital B has significant differences (*P* < 0.05), and hospital A has extremely significant difference (*P* < 0.01). See Table 5 [14–16].

## 5. Discussion

### 5.1. Monitoring and Childbirth

As can be seen from Table 1, the diagnosis rate of multi-independent groups in the two hospitals is higher, 75% and 83.33%, respectively, and the Cesarean section is 33.33% and 26.67%, respectively; but in the regular group the diagnosis rate is only 33.33% and 46.15%. The Cesarean section is higher, 100% and 83.33%. The percentage in the conventional group is much higher than that in the multi-indicator group, perhaps with doctors concerns that huge children may cause difficulty in production and maternal and child injury, especially the injury of brachial plexus neurons caused by shoulder dystocia. However, the literature reports that, due to the high accuracy of huge children and the literatism of the huge children, the choice of Cesarean section does not reduce the incidence of parent and child's complications, while Cesarean section may cause bleeding, infection, thrombosis outside the neonatal asphyxia, the existence of uterine scars, the uterus rupture, perforation, and so forth when the woman is prone to pregnancy and artificial abortion and the perforations. It is suggested that the clinician should accurately predict the situation of huge children. It is tightly observed on the production of huge children. When the production process is normal, try to choose vaginal delivery to ensure the safety of the mother and the child [17–19].

### 5.2. Umbilical Covered Neck Monitoring

As can be seen from Table 2, the diagnosis rate of multi-independent group in the two hospitals was 86.21% and 91.89%, and the Cesarean section rate was 36.00% and 29.41%; the regular group's diagnosed compliance rate was 38.46% and 36.84%, and, for Cesarean section, the yield has reached 80.00% and 71.43%, and the difference in Cesarean section rate in the two groups was significant (*P* < 0.05). The main reason for the significant increase in the number of cesarean section is that many pregnant women are afraid of fetal distress and abnormal production in the process of vaginal delivery, and obstetricians are also worried that there will be unexpected risks in the process of production, and umbilical cord around the neck is one of the indications of cesarean section. However, for pregnant women with umbilical cord around the neck without other indications of cesarean section, they should be encouraged to have a vaginal trial. It is not necessary to choose cesarean section because of a single umbilical cord around the neck. It is only necessary to consider cesarean section when umbilical cord around the neck affects production. In addition, adopting active and reliable premonitoring can not only effectively adjust the clinical progress of fetus with umbilical cord around neck but also have certain significance for reducing the current increasing rate of Cesarean section [20].

### 5.3. Fetal Distress Monitoring

As shown in Table 3, the cesarean section rates of the multi index groups in the two hospitals were 40.00% and 32.35%, respectively. The cesarean section rates in the routine group were 77.78% and 75.00%, respectively, which were higher than those in the multi-index group. However, the diagnostic coincidence rate in the multi-index group was higher than that in the routine group. The main reasons for the high cesarean section rate in the routine group are the inconsistent understanding of fetal distress by medical staff, different diagnostic scales, and the increasing probability of cesarean section due to fetal distress in order to avoid unnecessary medical disputes.

### 5.4. Detection of Cervical Length and Uterine Scar Thickness

Since the cervical length is an important indicator of cervical maturation, the vaginal measurement of the length of the cervix is a more objective ideal approach to a certain extent avoiding subjective factors. Through objective images and specific data measurements, the estimated results of traditional vaginal examination are replaced, and the vaginal ultrasound measurement does not require a filling bladder, and the display rate is 100%, so the cervical length can be used to predict delivery. Vaginal B-ultrasound was used in 25 cases and 31 cases in the two hospitals in this project, including 18 and 26 cases of cesarean section, accounting for 72.00% and 83.87%, which is similar to the situation reported in the literature. In addition, the measurement of the thickness of the uterine scar has a certain guiding effect on the decision of the pregnancy delivery. At present, in most cases, full-term pregnant women with scarred uterus mostly use cesarean section, and people with vaginal trial conditions rarely carry out vaginal trial, which is related to many medical disputes, tense medical relations, and limited technical level of medical personnel. Studies have shown that trial delivery is more favorable than cesarean section. For maternal women who become pregnant after Cesarean section, after the diabolic evidence of vaginal delivery, the opportunity to give trial production is to improve the success rate of vaginal delivery and reduce Cesarean section [21, 22].

## 6. Conclusions

In summary, reducing Cesarean section is a systematic project, and there is a significant need to adopt comprehensive interventions and even pay attention to the whole society. This paper makes a series of indicators for maternity, such as the monitoring of fetal size, cervical maturity, and uterine scar thickness, further reducing the risk of vaginal delivery, reducing misdiagnosis of Cesarean section, and thereby reducing Cesarean section. The results show that the total CNAXE rate was 48.10% and 39.29%, respectively, for 2018 and 2019, respectively, and the cesarean section of the conventional group was 65.75% and 63.64%. By contrasting data of multi-index group and conventional group, hospital B differences were significant (*P* < 0.05), and hospital A difference was extremely significant (*P* < 0.01). To a certain extent, the hospital should strengthen postpartum health publicity and education. Through the mission to let the mothers and their families understand the pros and cons of various production methods, the abnormal situation is found in time, timely treatment, and avoiding serious complications. Strengthening the psychological care of pregnant women in daily monitoring can make husband or relatives accompany pregnant women during childbirth and reduce pregnant women's fear, so that the production process is more smooth.

## Figures and Tables

**Table 1:** Comparison of the giant childhood delivery outcomes in each group of hospitals.

Unit	Group	Number of cases	Predicted value (cases)	Measured value (cases)	Coincidence rate (%)	Cesarean delivery (cases, %)	Vaginal delivery (cases, %)
Hospital B	Multiattribute group	79	12	9	75.00	3 (33.33)	6 (66.67)
	Regular group	73	9	3	33.33	3 (100.00)	0 (0.00)

Hospital A	Multiattribute group	84	18	15	83.33	4 (26.67)	11 (73.33)
	Regular group	77	13	6	46.15	5 (83.33)	1 (16.67)

**Table 2:** Comparison of the endings of the two hospitals in each group.

Unit	Group	Number of cases	Predicted value (cases)	Measured value (cases)	Coincidence rate (%)	Cesarean delivery (cases, %)	Vaginal delivery (cases, %)
Hospital B	Multiattribute group	79	29	25	86.21	9 (36.00)	16 (64.00)
	Regular group	73	13	5	38.46	4 (80.00)	1 (20.00)

Hospital A	Multiattribute group	84	37	34	91.89	10 (29.41)	24 (70.59)
	Regular group	77	19	7	36.84	5 (71.43)	2 (28.57)

**Table 3:** Comparison of the fetches of the fetuses of the two hospitals.

Unit	Group	Number of cases	Predicted value (cases)	Measured value (cases)	Coincidence rate (%)	Cesarean delivery (cases, %)	Vaginal delivery (cases, %)
Hospital B	Multiattribute group	79	39	35	89.74	14 (40.00)	21 (60.00)
	Regular group	73	28	9	32.14	7 (77.78)	2 (22.22)

Hospital A	Multiattribute group	84	37	34	91.89	11 (32.35)	23 (67.65)
	Regular group	77	19	8	42.10	6 (75.00)	2 (25.00)

**Table 4:** Comparison of the outcomes of the two hospitals in various groups of orientation (example).

Unit	Group	Number of cases	Occiput anterior position		Occiput transverse position		Occiput posterior position		Caesarean delivery rate (%)
			Cesarean delivery	vaginal delivery	Cesarean delivery	vaginal delivery	Cesarean delivery	vaginal delivery	
Hospital B	Multiattribute group	79	12	37	14	5	10	1	48.10
	Regular group	73	26	17	17	8	5	0	65.75

Hospital A	Multiattribute group	84	13	32	15	14	5	5	39.29
	Regular group	77	25	14	10	10	14	4	63.64

**Table 5:** Comparison of two hospital delivery outcomes (example, %).

Unit	Group	Number of cases	Cesarean delivery	Vaginal delivery	*χ*2 value	*P* value
Hospital B	Multiattribute group	79	38 (48.10)	41 (51.90)	4.812	<0.05
	Regular group	73	48 (65.75)	25 (34.25)		

Hospital A	Multiattribute group	84	33 (39.29)	51 (60.71)	9.532	<0.01
	Regular group	77	49 (63.64)	28 (36.36)		

## Data Availability

The data used to support the findings of this study are available from the corresponding author upon request.
